# Enhanced Methodology and Experimental Research for Caged Chicken Counting Based on YOLOv8

**DOI:** 10.3390/ani15060853

**Published:** 2025-03-16

**Authors:** Zhenlong Wu, Jikang Yang, Hengyuan Zhang, Cheng Fang

**Affiliations:** 1 College of Engineering, South China Agricultural University, Guangzhou 510642, China; zhenlong.wu@kuleuven.be (Z.W.); yjkscau@stu.scau.edu.cn (J.Y.); 20223175022@stu.scau.edu.cn (H.Z.); 2 Faculty of Bioscience Engineering, Katholieke Universiteit Leuven (KU LEUVEN), Kasteelpark Arenberg 30, 3001 Leuven, Belgium; 3 State Key Laboratory of Livestock and Poultry Breeding, South China Agricultural University, Guangzhou 510642, China

**Keywords:** chicken counting, layered cage farming, object detection, poultry, precision livestock farming

## Abstract

Accurately counting chickens in densely packed cages is a major challenge in large-scale poultry farms. Traditional manual counting methods are labor-intensive, costly, and prone to errors due to worker fatigue. Furthermore, current deep learning models often struggle with accuracy in caged environments because they are not well-equipped to handle occlusions. In response, we propose the You Only Look Once-Chicken Counting Algorithm (YOLO-CCA). YOLO-CCA improves the YOLOv8-small model by integrating the CoordAttention mechanism and the Reversible Column Networks backbone. This enhancement improved the YOLOv8-small model’s *F1* score to 96.7% (+3%) and average precision_50:95_ to 80.6% (+2.8%). Additionally, we developed a threshold-based continuous frame inspection method that records the maximum number of chickens per cage with corresponding timestamps. The data are stored in a cloud database for reliable tracking during robotic inspections. The experiments were conducted in an actual poultry farming environment, involving 80 cages with a total of 493 chickens, and showed that YOLO-CCA raised the chicken recognition rate to 90.9% (+13.2%). When deployed on a Jetson AGX Orin industrial computer using TensorRT, the detection speed increased to 90.9 FPS (+57.6 FPS), although the recognition rate slightly decreased to 93.2% (−2.9%). In summary, YOLO-CCA reduces labor costs, improves counting efficiency, and supports intelligent poultry farming transformation.

## Simple Summary

The system developed in this study provides a practical, real-time machine vision solution to address the low efficiency and high cost of manual chicken counting. By integrating enhanced deep learning models with low-cost, edge-based computing and storing data in a cloud database, the system can automatically track chicken flock sizes throughout the day. While adapting the algorithm for industrial hardware may result in a slight accuracy trade-off, the benefits of speed and continuous data collection are substantial, enabling more efficient management and health monitoring. In the future, we will continue refining the method to accommodate various cage designs, incorporate additional health indicators, and further reduce computational requirements that can increase its practicality.

## 1. Introduction

Livestock farming has transformed from traditional manual labor to a modern, efficient industry, significantly influenced by digital advancements, particularly precision livestock farming (PLF) [1,2,3]. While the egg poultry industry in the United States and Europe is gradually adopting cage-free systems, caged husbandry remains predominant in China due to its large population and the necessity for efficient food supply through large-scale caged breeding [4,5,6]. Accurate chicken counting in caged environments is crucial because it reduces labor costs, improves farm management efficiency, and provides early warnings when the number of chickens in a cage unexpectedly decreases, which can indicate issues such as disease or other welfare concerns.

PLF emerges as a promising solution, integrating sensor technology, machine learning, and other advanced methodologies to monitor and optimize the health, behavior, and production benefits of animals in real-time [7,8,9]. Among its key tasks is chicken detection, aiming to automatically locate and identify chickens based on their visual appearance and behavioral characteristics [10,11]. Although deep learning techniques have improved the accuracy and efficiency of chicken counting [12,13,14], most of these methods are designed for free-range conditions and do not fully address the unique challenges of caged environments, such as dense occlusions, limited space, and environmental noise [15]. This gap in existing solutions can delay management decisions and lead to higher operational costs.

To address the issues, this study developed a laying hens inspection algorithm named You Only Look Once-Chicken Counting Algorithm (YOLO-CCA), focusing primarily on the precise counting of live chickens in each cage. The existing literature has shown that integrating attention mechanisms or replacing the backbone can effectively enhance model detection efficiency in poultry farming [16,17,18]. However, the optimization effects achieved by introducing attention mechanisms and backbone replacement in caged chicken detection have not been experimentally validated. Therefore, we compared various mainstream attention mechanisms and backbones, quantified the improvements, and selected Reversible Column Networks (RevCol) and CoordAttention (CoordATT) as the best combination. The YOLO-CCA enhances detection accuracy by replacing the YOLOv8-small (YOLOv8s) backbone with RevCol and incorporating the CoordATT mechanism. This algorithm is complemented by a threshold-based frames analysis that accurately processes video streams from robotic inspections, ensuring precise counts of chickens in each cage. By uploading inspection data to a cloud database, this algorithm enables managers to filter and process data effectively. The method emphasizes monitoring and recording fluctuations in chicken numbers within cages, indirectly identifying deceased chickens, thus overcoming the limitations of direct detection technologies and offering a more efficient, cost-effective solution [19,20,21].

The remainder of this paper is organized as follows. Section 2 reviews related work on poultry detection, attention mechanisms, and backbone replacement strategies. Section 3 describes the proposed YOLO-CCA method, including its network architecture and frame analysis approach. Section 4 presents experimental results from tests conducted in real poultry farming environments. Section 5 discusses the experimental findings and their implications, and Section 6 summarizes the paper and outlines future research directions.

## 2. Related Works

### 2.1. Existing Chicken Detection Technologies

The introduction of AI, particularly deep learning technology, has infused modernization into the livestock industry [22,23,24]. Current detection technologies for caged chickens mainly concentrate on two key areas: correct identification of live chickens and timely detection of deceased chickens. Hao et al. developed a dead broiler detection system based on the YOLOv3 network tailored for large-scale poultry farms in China. Although this system effectively reduced the workload of breeders, its static inspection approach, as opposed to mobile, could not leverage multi-frame analysis to mitigate occlusion effects, and the scarcity of dead chicken datasets led to missed and false detections [25]. Concurrently, Luo et al. focused on the automatic detection of dead laying hens by combining NIR, TIR, and depth images for multi-source image registration, enhancing the model’s accuracy and robustness. However, the low processing efficiency and high equipment costs did not meet real-time detection needs [26]. Additionally, Yang et al. proposed a deep learning-based defencing algorithm for effectively identifying and restoring images of chickens in cages, but it also faced efficiency and cost challenges, with its complex GAN network and high equipment requirements difficult to apply and deploy on actual industrial control machines [27]. Although these studies have made technological progress, they still face limitations, particularly in terms of efficiency and practicality.

### 2.2. Application of Attention Mechanisms in Animal Detection

Attention mechanisms work by assigning greater weight to pivotal pixels or regions, thus focusing the model more intently on the target, leading to enhanced detection precision. Regarding attention mechanisms, we employed prevalent techniques, such as ShuffleAttention (SA), Efficient Multi-Scale Attention (EMA), Spatial Groupwise Enhance (SGE), and Simple Attention Module (SimAM), to evaluate the degree of enhancement CoordATT offers to the target model.

SA, as proposed by Zhang et al., hinges on the core idea of reducing computational load through the incorporation of group convolutions. It then applies spatial and channel attention to each group before facilitating intergroup information exchange to boost computational efficiency and generalization capability [28]. EMA, put forth by Ouyang et al., aims to retain information in every channel while minimizing computational overhead. It achieves this by dimensionally reducing certain channels and grouping them into multiple sub-features, ensuring an equitable spatial semantic feature distribution across each feature set [29]. SGE, as conceptualized by Li et al., can adjust the importance of each sub-feature to enhance the feature representation of complex objects [30]. SimAM, introduced by Yang et al., enhances the representation capability of convolutional networks by inferring 3D attention weights for feature maps [31]. The CoordATT mechanism, introduced by Hou et al., is an innovative mobile network attention mechanism designed by embedding positional information into channel attention [32]. In this approach, the input feature map is processed with two separate global pooling operations along the height and width dimensions. These operations produce two sets of direction-specific descriptors that capture spatial dependencies along each axis. The descriptors are then concatenated and passed through convolution layers combined with non-linear activations to generate attention weights for each channel. These weights modulate the original feature map, allowing the network to adjust channel responses based on spatial positions.

To assess the effectiveness of various attention mechanisms, our experiments systematically evaluated the impact of CoordATT against other techniques, confirming its superiority as the chosen attention mechanism for enhancing the object detector tailored to fence and poultry features.

### 2.3. Impact of Backbone Network Replacement on Model Performance

Backbone replacement in deep-learning architecture plays a pivotal role in enhancing model adaptability and performance across varied detection environments. In the aspect of backbone augmentation, we engaged with mainstream architecture, such as Large Selective Kernel Network (LSKNet), Residual Network 18 (ResNet18), Faster Neural Networks (FasterNet), Codesigning and Scaling ConvNets (ConvNeXtV2), and EfficientFormerV2, to replace the original network and validate the degree of performance uplift.

LSKNet, as described by Li et al., is capable of dynamically adjusting its large spatial receptive field, allowing it to better model the diverse scale backgrounds in various object scenes [33]. ResNet18, introduced by He et al., is a variant in the ResNet series containing 18 layers. Its salient feature is the introduction of residual connections, allowing features from preceding layers to bypass certain layers and be summed with features of subsequent layers [34]. FasterNet, delineated by Chen et al., efficiently extracts spatial features by introducing a novel partial convolution approach, simultaneously reducing redundant computations and memory accesses [35]. ConvNeXtV2, as proposed by Woo et al., bolsters the performance of pure ConvNets across various recognition benchmarks by synergizing self-supervised learning techniques with architectural advancements [36]. EfficientFormerV2, delineated by Li et al., re-examines the design choices of visual transformers and introduces an optimized super-network to achieve low latency and high parameter efficiency, resulting in performance comparable to MobileNet in terms of size and speed [37].

Propounded by Cai et al., the RevCol network comprises multiple subnetworks and adopts multilevel reversible connections between them [38]. Each subnetwork contains reversible blocks designed so that the input can be recovered from the output. In practice, a reversible block splits the input feature map into two parts, applies transformation functions to one part, and then combines the results in an additive manner. This reversible design ensures that the information is preserved as features pass through each block, rather than being compressed or lost. Additionally, multiple columns in the network are connected with reversible links at different feature levels, which helps maintain detailed information throughout the processing stages. In our study, we implement the RevCol network following the design proposed by Cai et al., and we apply it to object detection tasks where it contributes to improved performance by retaining important feature details.

## 3. Materials and Methods

### 3.1. Ethics Approval

All experiments were performed according to regulations and guidelines established by the experimental animal administration and ethics committee of South China Agricultural University (SCAU#SYXK-2019-0136).

### 3.2. Acquisition of Datasets

This section outlines the methodology employed for the systematic collection of the dataset used in this study, crucial for training and evaluating the YOLO-CCA model. The dataset was carefully gathered to represent the real-world conditions of a caged chicken farm, ensuring the accuracy and applicability of the model in agricultural settings.

The dataset utilized in this study was sourced from a caged layer chicken farm located in Raoping County, Chaozhou City, Guangdong Province. The chicken house was divided into two sides, each containing four layers of cages. A camera (Siyi A8 mini, China) equipped with mechanical stabilization, was used for data collection. To ensure the camera’s field of view covered two railings simultaneously without including a third (ensuring that only one full cage appeared in the field of view), the photographing distance was controlled to approximately 50 cm. This setup enabled comprehensive coverage of individual cages without overlapping with adjacent ones. The camera was mounted on the adjustable column side of the inspection robot, tasked with monitoring a row of chicken cages. The Pan Tilt’s stabilization effectively counteracted vibrations caused by the robot’s movement, ensuring high-quality video streams.

**Figure 1:**
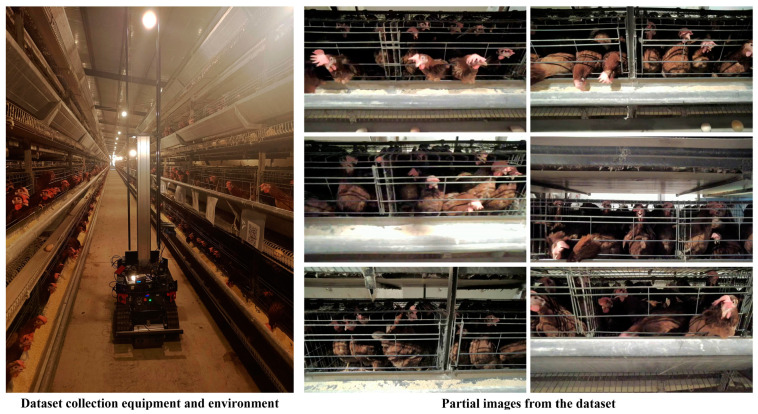
Dataset collection environment and partial inspection images.

After obtaining permission from the cooperating enterprise, data collection was scheduled for 26 July 2023, between 6:00 AM to 11:00 AM. This time frame was sufficient for the robot to traverse every aisle of the chicken house and collect a substantial amount of data. At robot startup, the industrial control computer automatically executed the camera and recording code scripts. The camera resolution was set to 1280 × 720 pixels, with capture angles varying between 0° and −45° (ensures diversity of viewpoint data), and a rate of 30 Frames Per Second (FPS). The dataset comprises 14 inspection video segments. By extracting one frame every 10 frames, a total of 6500 images were selected. The studied chicken breed was the Guangdong Yellow Feather layer chicken. Each cage, measuring 40 cm × 60 cm × 40 cm, held 5 to 8 chickens which are 40 to 50 weeks old. To ensure image quality, the inspection robot’s speed was set at 0.2 m per second. The industrial computer automatically activated the camera and recording software upon robot startup, enabling the robot to traverse each aisle completely and collect a sufficient amount of data. Figure 1 displays the on-site environment of dataset collection and samples of the images.

### 3.3. Data Pre-Processing

This section describes the data pre-processing steps undertaken to ensure the quality and utility of the dataset for training the object detection model. The focus was on refining the dataset to accurately represent chicken and cage characteristics, facilitating effective model training and evaluation.

The collected data were first screened to exclude any abnormal data due to accidental captures or other reasons. In the annotation process, our emphasis was on labeling the chickens in the dataset and the fences of their cages to support the object detection task of this study.

Given that the number of chickens in each cage was substantial and they might be crowded together, we chose to annotate only the chicken’s heads, which is a robust indicator of live chickens [39]. The chicken head region is defined to include the comb with the front facial area serving as the boundary. In instances where a chicken was engaged in pecking and its head was partially occluded, the annotation extended to include a portion of the neck and the visible area near the feeding trough that was indicative of the head region. While this might have increased the risk of missed detections, it significantly reduced the chances of false positives.

**Figure 2:**
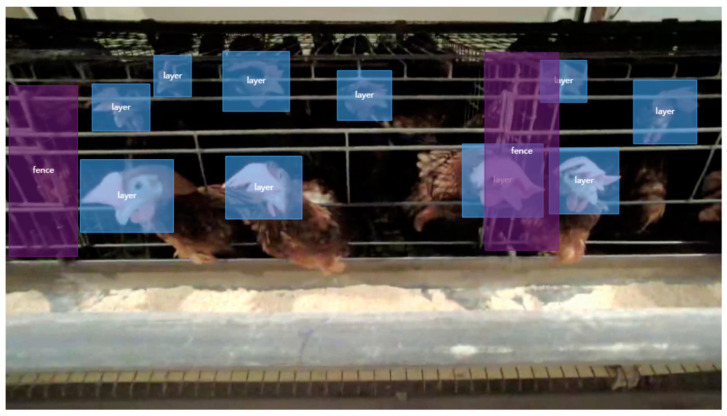
Labeled images of chickens and fences.

The annotations were carried out using the EasyDL platform based on Baidu’s PaddlePaddle [40], and the obtained VOC format dataset was manually converted to a format suitable for YOLO for algorithm training and testing. To evaluate model performance, the dataset was randomized and split into training, validation, and test sets in an 8:1:1 ratio, resulting in 5200 images for training and 650 images each for validation and testing. The labeling management interface is shown in Figure 2. In the EasyDL system, bounding boxes are manually drawn and a subset of chicken images are classified to train an automatic annotation model, which is then used to perform further automated labeling that is subsequently verified by inspectors. Additionally, it is important to note that although lighting intensity varies, the shallow depth of the cages ensures that humans can accurately count the number of chickens within each cage, providing a reliable reference for experimental comparisons.

### 3.4. Overall Process Flow Diagram

This section delineates the comprehensive process flow used in this study to automate live chicken counting and monitoring within poultry farms.

**Figure 3:**
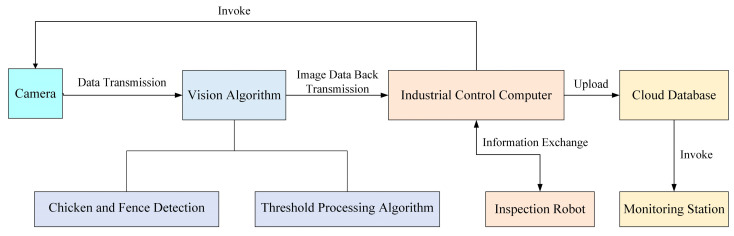
Comprehensive Workflow of the Automated Chicken Counting and Monitoring System.

Figure 3 illustrates this process framework, which initiates when cameras mounted on inspection robots capture video streams of caged chickens. These streams undergo a series of image processing steps where advanced algorithms detect live chickens and distinguish between different cages. This multi-step process not only counts chickens but also analyzes time-series data and frame changes to accurately determine each cage’s spatial location, enhancing data precision and reliability.

**Figure 4:**
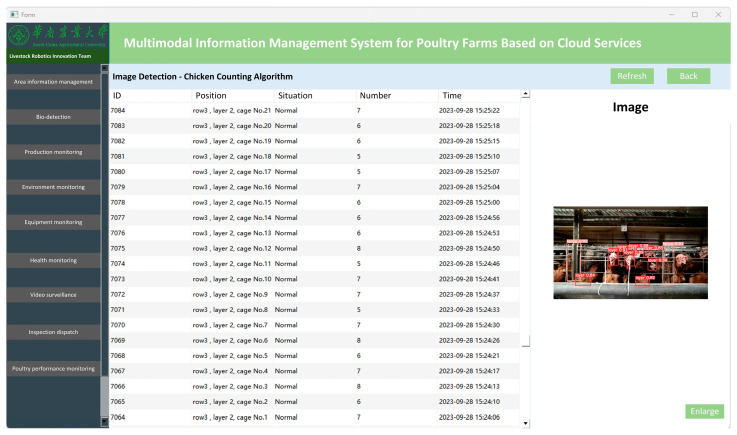
The interface of the management system.

The interface of the management system is shown in Figure 4. The data are processed locally and, once validated, are transmitted via a robust local network setup involving multiple access points to the Alibaba Cloud Server for storage and further analysis (link: https://github.com/alibaba/AliSQL, accessed on 28 September 2023). At the monitoring station, managers have access to this real-time data through a custom-developed poultry management system, allowing immediate responses to changes such as a decline in chicken numbers, potentially indicating mortality [41]. The system’s ability to quickly detect and alert managers about such events is crucial for maintaining the health and productivity of the flock.

### 3.5. Overall Flow of the Visual Algorithm

**Figure 5:**
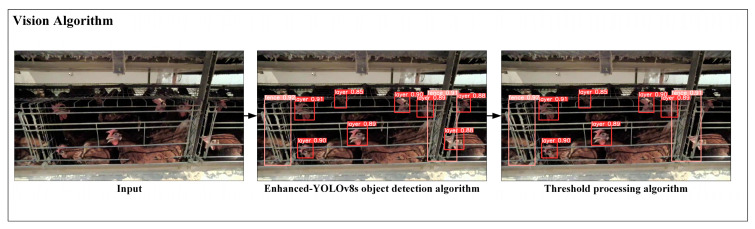
Flowchart illustrating the two-step process of the caged chicken counting algorithm: object detection followed by threshold processing.

In this paper, the caged chicken counting algorithm is mainly divided into two parts: object detection and threshold processing. First, the video stream captured by the camera is input into the enhanced YOLOv8s object detection model for analysis, aiming to identify the categories of chicken heads and fences and determine pixel coordinate points. Subsequently, the data generated from image recognition undergo threshold processing. This algorithm determines and filters out all chickens within a target cage by iterating through the coordinates of fences and chicken heads, finally outputting the desired results. Figure 5 illustrates the overall process of the chicken counting algorithm.

### 3.6. Introduction of the Object Detection Algorithm

This section introduces the object detection algorithm essential for the poultry inventory system, focusing on the implementation and enhancement of the YOLOv8 series. Detailed explanations are provided on how YOLOv8 adapts to the challenges of accurately detecting and classifying objects within a poultry farm environment.

The object detection component plays a pivotal role in the poultry inventory algorithm, serving as the key in identifying and localizing both the fences and chicken heads. YOLO stands as an end-to-end object detector renowned for its ease of training, delivering high performance in terms of speed and accuracy. Through its end-to-end approach, the YOLO series segments and localizes objects within images, subsequently predicting the objects’ categories and positions. Multiple iterations of YOLO have been released, among which YOLOv8 is an advanced version. Developed by Ultralytics, YOLOv8 stands as a continuation of the YOLOv5’s original team [42]. The notable flexibility of this model lends itself to further enhancements in our study. The source code and architecture are referenced from https://github.com/ultralytics/ultralytics (accessed on 6 September 2023). Given the requirement for the poultry inventory algorithm to be deployed in the industrial computer of robots, we selected the lightweight versions of YOLOv8s as the initial object detection algorithm models.

**Figure 6:**
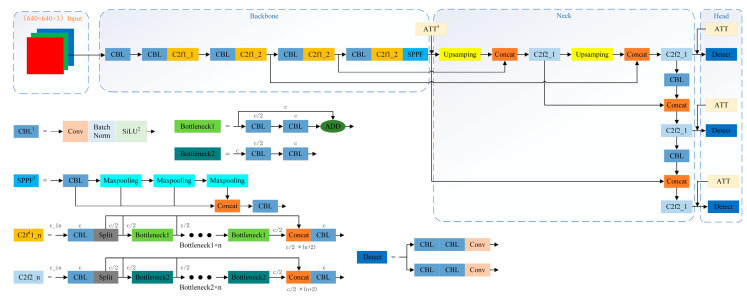
Detailed network structure of YOLOv8s used for chicken and fence object detection. Note: CBL = Convolution with Batch Normalization and SiLU; SiLU = Sigmoid Linear Unit; SPPF = Spatial Pyramid Pooling Fusion; C2f = CSPBottleneck with 2 convolutions; ATT = Attention Module.

Figure 6 illustrates the detailed network structure of YOLOv8s, which is used for detecting both chicken heads and cage fences. In our approach, attention mechanisms are inserted between the backbone and the neck to emphasize key image features, thereby enhancing detection accuracy in complex environments. Additional attention modules are incorporated within the detection head to improve the model’s sensitivity to critical regions, such as distinct parts of the chicken, which reduces false-positive detections.

To validate that introducing attention mechanisms and backbone replacement can enhance the efficiency and accuracy of feature extraction, this paper individually substitutes the original YOLOv8s backbone network with LSKNet, RevCol, ResNet18, FasterNet, ConvNeXtV2, and EfficientFormerV2. At the end of the backbone and the start of the head, attention mechanisms such as SA, EMA, SGE, SimAM, and CoordATT are integrated. The effectiveness of these modifications was tested in practical experiments.

### 3.7. Introduction of the Threshold Processing Algorithm

This section discusses the development and implementation of a threshold processing algorithm designed to enhance the accuracy of chicken detection by refining results from the initial detection phase. The algorithm specifically addresses potential errors from overlapping cages and optimizes the count of chickens, contributing to the reliability of our model in operational settings. A key advantage of this approach is the detection of fences using two bounding boxes rather than a single bounding box for each cage. This method ensures that the resultant bounding box for each cage is complete and accurate, minimizing errors associated with incomplete or overlapping cage images.

**Figure 7:**
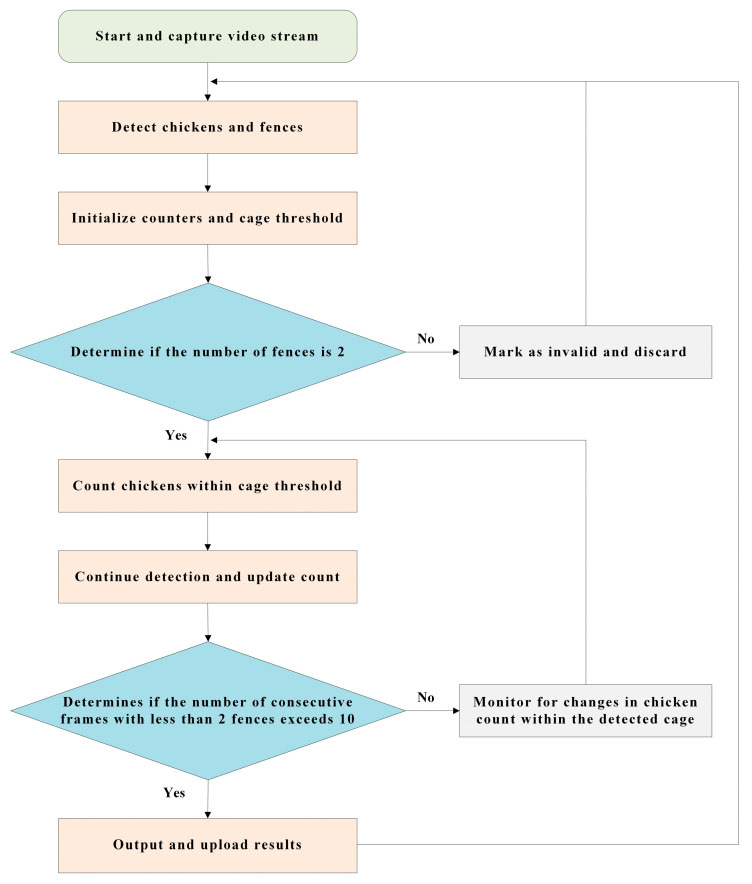
Flow chart of the thresholding algorithm for refining chicken detection inside cages.

To eliminate errors that may arise from detecting chickens in adjacent cages, we introduced a post-processing step to further refining the detection results, ensuring the reliability of our model in real-world scenarios. Following the primary detection, a threshold processing stage was incorporated, which not only utilized the spatial distribution of the cages and chicken heads to further filter and tally detected targets but also actively monitored and updated the maximum count of chickens within individual cages. The process flow of the threshold processing segment is depicted in Figure 7.

Initially, the algorithm identifies the bounding boxes of fences and chicken heads from all detected objects and extracts their horizontal coordinates. These coordinates provide the basis for determining the cage location and selecting chickens in subsequent steps. Subsequently, the algorithm calculates the minimum and maximum horizontal coordinates for each fence bounding box, thereby defining the lateral spatial threshold of the cage.

Taking into account the dynamic variation in fence numbers, when the count stabilizes at two, the algorithm activates the counting process to update the maximum number of chickens in real-time. It compares the horizontal coordinates of detected chicken head bounding boxes against the lateral spatial thresholds of the cage; if a chicken head is located within the range of the cage, the count of chickens within the cage is updated. The subsequent counting logic accounts for dynamic changes in fence numbers, activating the real-time update of maximum chicken counts when the count stabilizes at two. If the fence count temporarily drops to one, the algorithm performs a continuous frame check. Should fewer than ten consecutive frames detect a single fence, it is adjudicated as a false detection, and the original maximum chicken count is maintained. If the consecutive frames exceed ten, the algorithm concludes that the cage has been passed, and thus outputs the maximum chicken count and detection time for that cage. These temporal data can be utilized in conjunction with the robot’s coordinates at that time to locate the cage position within the cloud database. Concurrently, the algorithm selects and outputs the image with the highest number of chickens within the cage. Consequently, each cage’s maximum chicken count and temporal information, along with key images, are preserved and uploaded to the cloud database for managerial evaluation and further analysis.

### 3.8. Algorithm Deployment

This section outlines the steps involved in deploying the object detection algorithm, from model conversion to its integration into robotic systems. Special emphasis is placed on the transition of the model into formats suitable for real-time application, ensuring efficiency and reliability in a production environment.

**Figure 8:**
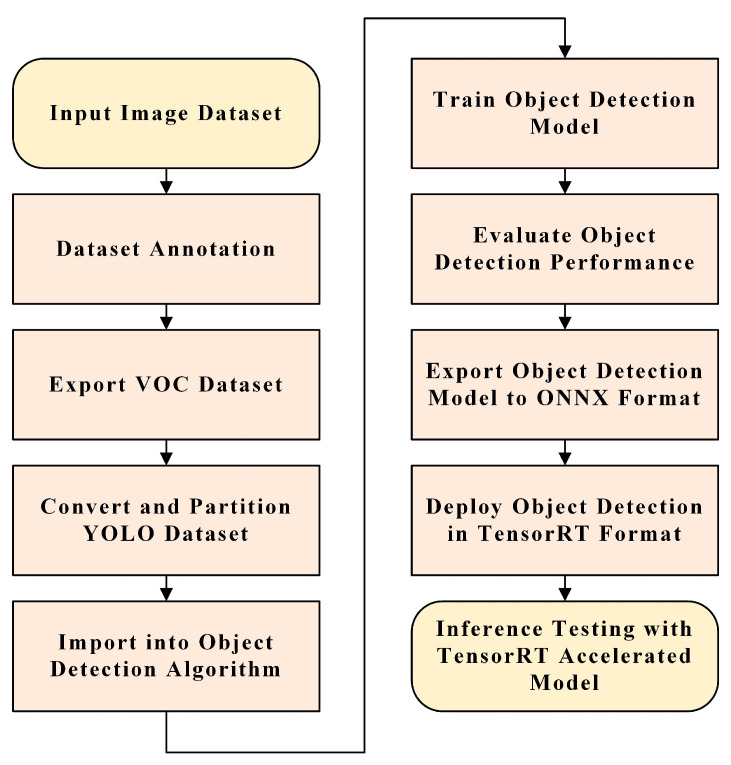
Comprehensive flowchart of model training, conversion, and deployment for efficient integration.

To facilitate the deployment of the algorithm onto robots, the object detection model was initially converted into the Open Neural Network Exchange (ONNX) format, subsequently transitioning this ONNX model into the TensorRT format. TensorRT is an efficient runtime library tailored for low computational power and energy efficiency, enhancing the model’s operational efficiency. During deployment, the NVIDIA Jetson Orin AGX embedded system was utilized. NVIDIA’s Jetson series is specifically designed for high-performance applications in AI and neural network projects. Its native Jetpack package seamlessly addresses the requirements for deep learning environments, making project deployment straightforward [43]. Figure 8 delineates the entire model training and deployment process.

### 3.9. Evaluation Criteria

To assess the performance of the object detection algorithm presented in this study, several metrics were employed, including precision (*P*), recall (*R*), *F1* score, average precision_50:95_ (AP_50:95_), FPS, and parameters. *P* gauges the model’s accuracy of predictions, with higher values indicating increased accuracy. *R* measures the instances that the model failed to detect, with higher values indicating a reduced rate of missed detections. The *F1* score evaluates the balance between the model’s *P* and *R*, where values closer to 1 signify superior model performance. AP_50:95_ represents the mean accuracy across different intersections over union (IoU) thresholds (ranging from 0.50 to 0.95), with higher values indicating enhanced performance. FPS indicates the speed at which the model processes data, with higher values indicating faster processing speeds. Both parameters and model size serve as crucial indicators of model scale, with parameters representing the total count of internal model parameters and model size reflecting the storage space occupied by the model on the embedded system. Smaller values suggest a more compact and lightweight model.

*P* is the ratio of samples correctly predicted as true positive (*TP*) to all samples predicted as positive, including both *TP* and false positive (*FP*). A higher value of *P* indicates a higher prediction accuracy, as depicted in (1):

(1) $$P=\frac{TP}{TP+FP}.$$

*R* is the ratio of samples correctly predicted as *TP* to all actual positive samples, encompassing both *TP* and false negative (*FN*). Higher *R* values indicate reduced missed detections, as depicted in (2):

(2) $$R=\frac{TP}{TP+FN}.$$

The *F1* score metric is the harmonic meaning of *P* and *R*, placing equal importance on both while evaluating the model’s performance. Scores closer to 1 indicate that both *P* and *R* are relatively high, as depicted in (3):

(3) $$F1=2\cdot \frac{P\cdot R}{P+R}$$

AP represents the proportion of correctly predicted positive samples, with higher proportions indicating fewer detection errors. AP_50_ refers to the average *P* at an IoU of 0.5, while AP_50:95_ represents the mean AP across ten IoU thresholds (from 0.5 to 0.95 in increments of 0.05).

FPS indicates the number of image frames the target model can process per second.

Additionally, for the performance evaluation of threshold processing and algorithm deployment, we established three metrics: sample selection rate, chicken selection rate, and chicken recognition rate.

Sample selection rate: This is calculated as the count of samples post-threshold processing divided by the complete cage sample count. This metric reflects the capacity to extract relevant samples from the original data. The formula is depicted in (4):

(4) $$\mathrm{Sample}\;\mathrm{selection}\;\mathrm{rate}\;=\frac{\mathrm{Sample}\;\mathrm{count}\;\mathrm{after}\;\mathrm{threshold}\;\mathrm{processing}}{\mathrm{Sample}\;\mathrm{count}\;\mathrm{with}\;\mathrm{complete}\;\mathrm{cage}}$$

Chicken selection rate: This metric measures the ability to screen valid chickens in a single cage from all chickens within the picture. It is calculated as the number of chickens in a single cage after thresholding divided by the number of chickens detected in the image. The formula is depicted in (5):

(5) $$\mathrm{Chicken}\;\mathrm{selection}\;\mathrm{rate}\;=\frac{\mathrm{Chicken}\;\mathrm{count}\;\mathrm{after}\;\mathrm{threshold}\;\mathrm{processing}}{\mathrm{Detected}\;\mathrm{chicken}\;\mathrm{count}}$$

Chicken recognition rate: This metric is used to evaluate the performance of the deployed algorithm in detecting chickens in the cage. It is calculated as the number of chickens detected in the cage divided by the actual number of chickens after manual verification. The formula is depicted in (6):

(6) $$\mathrm{Chicken}\;\mathrm{recognition}\;\mathrm{rate}\;=\frac{\mathrm{Detected}\;\mathrm{chicken}\;\mathrm{count}}{\mathrm{Actual}\;\mathrm{chicken}\;\mathrm{count}}$$

### 3.10. Experimental Setting

For this research, model training was conducted using an Intel(R) Core(TM) i7-3960X CPU @ 3.30 GHz (Santa Clara, California, USA), 16 GB RAM, and a GeForce GTX 1080Ti 11 G GPU (Santa Clara, California, USA). Our operating system was Windows 11 Professional, with a software environment comprising Python 3.9, Pytorch 1.12.0, CUDA 11.3, CUDNN 8.4.1, Visual Studio 2019, and TensorRT 8.4.1.

To ensure reproducibility and robust model performance, our deep learning training pipeline included several key components. First, the raw dataset was preprocessed using standard data augmentation techniques, such as random flipping, scaling, and color jittering. Input images were normalized and resized to maintain consistent dimensions, which helped improve model generalization. We then employed the YOLOv8s architecture as the base model and incorporated the necessary modifications for the YOLO-CCA framework. Hyperparameters were tuned via an iterative grid search process over candidate values. The final training settings are summarized in Table 1.

Specifically, a batch size of 32 was used, and the model was trained for 300 epochs, resulting in a total of 60,938 iterations. An initial learning rate of 1 × 10^−4^ was selected, with adjustments made based on the outcomes of 100 rounds of iterative training and debugging. A learning rate scheduler (e.g., cosine annealing or step decay) was implemented to gradually reduce the learning rate over time, thereby preventing premature convergence or oscillations. Additionally, model checkpoints were saved at regular intervals, and the best-performing model was selected based on validation metrics, such as *F1* score and AP_50:95_. Early stopping criteria were also applied to avoid overfitting if validation loss did not improve over a set number of epochs.

For optimization, we consistently employed the widely used Stochastic Gradient Descent (SGD) optimizer with momentum. During training, the loss function was decomposed into several components, including classification loss, bounding box regression loss, and distribution focal loss, ensuring balanced gradient updates across all aspects of the object detection task. Training and validation metrics were logged in real time using TensorBoard, enabling continuous monitoring of model performance and facilitating detailed post-training analysis.

## 4. Results

### 4.1. Enhancement in Detection Efficacy Using the Enhanced YOLOv8s Model

In the original YOLOv8s model, a novel attention mechanism was investigated, followed by comprehensive training and evaluation on the test set. The test results are presented in Table 2.

Regarding model size, the majority of models integrating attention mechanisms only introduced a minimal increase in parameters compared to the original YOLOv8s. For instance, the sizes of YOLOv8s+SA and YOLOv8s+SGE are almost identical to the benchmark model. On the other hand, YOLOv8s+EMA and YOLOv8s+CoordATT saw negligible increases of 0.2 MB and 0.14 MB, respectively. Such trivial augmentations should be deemed acceptable during model deployment, especially considering their performance enhancements.

The above analysis indicates the superior performance of YOLOv8s+CoordATT across multiple evaluation metrics. Among all the attention-integrated models, its overall efficacy was paramount. Particularly, the pronounced enhancement in the *F1* score reflects its exceptional prowess in balancing *P* and *R*. These findings have motivated the use of CoordATT as the attention mechanism of choice for subsequent work.

In Table 3, YOLOv8s+CoordATT+RevCol performs best in *P*, *F1* score, AP_50:95_, and processing speed while also having the fewest parameters and smallest model size. This makes it the optimal choice in terms of performance, speed, and efficiency. Therefore, we chose YOLOv8s+CoordATT+RevCol as the foundation for subsequent research.

Table 4 shows the comparative analysis of statistical outcomes among different algorithms, it was observed that each variant contributed to performance in a distinct manner. Although YOLOv7-tiny exhibited superior accuracy, its AP_50:95_ metric was slightly lower than that of YOLOv8s. YOLOX-s demonstrated a slight increase in accuracy over the original YOLOv8s but experienced a decline in other critical metrics. Within the YOLOv8s variants, the YOLOv8s+CoordATT achieved enhanced performance in both accuracy and *F1* score, while YOLOv8s+RevCol outperformed in recall rate, *F1* score, and AP_50:95_.

The combination of YOLOv8s+CoordATT+RevCol excelled across all assessment metrics, confirming the synergistic effect of the CoordATT and RevCol technologies in performance enhancement.

**Figure 9:**
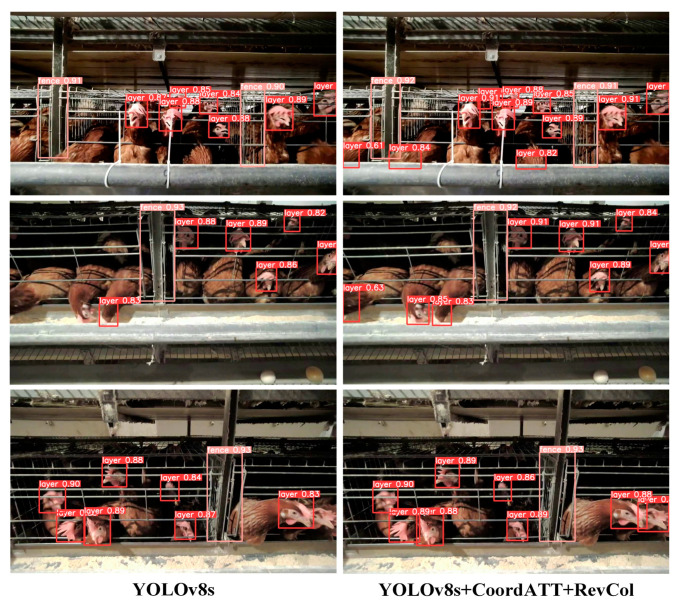
Comparative visualization of object detection results between the original YOLOv8s and the YOLOv8s+CoordATT+RevCol.

Beyond individual performance improvements, the integrated application of YOLOv8s+CoordATT+RevCol marked a significant advancement in both precision and detection efficiency. This variant emerged as the preferred option for the YOLO-CCA target detection module, outshining the original model in detection accuracy and reducing omissions, as illustrated in Figure 9. Figure 9 highlights the superiority in detection accuracy and reduction in missed detections.

**Figure 10:**
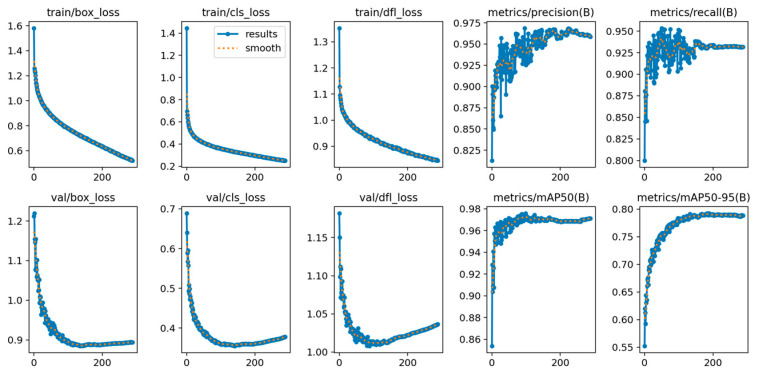
Performance metrics change curve of YOLO-CCA model during training.

Figure 10 presents a comprehensive view of the training dynamics and validation performance of the YOLO-CCA model over 300 epochs. The key metrics illustrated include losses for bounding boxes, classification, and distribution focal loss, alongside *P*, *R*, and mean average precision (mAP) scores.

a. Loss Metrics: All loss metrics (train/box_loss, val/box_loss, train/cls_loss, val/cls_loss, train/dfl_loss, val/dfl_loss) consistently decrease over the training epochs, illustrating effective learning in object localization, classification accuracy, and bounding box corner precision. The declining trend across both training and validation datasets indicates that the model generalizes well without overfitting, which is a positive sign of robust model training.

b. Precision and Recall Metrics: After initial volatility, both precision and recall metrics (metrics/precision(B), metrics/recall(B)) improve and stabilize as the training progresses. This stabilization at high levels suggests that the model has effectively minimized both false positives and false negatives, achieving a reliable balance critical for practical deployment in object detection tasks.

c. mAP Scores: The average precision scores at different IoU thresholds (mAP50 and mAP50-95) show a steady increase, reaching a plateau towards the end of the training process. This indicates not only the model’s precision in detecting objects accurately but also its ability to maintain this accuracy across a range of IoU thresholds, which is crucial for robust performance in varied real-world scenarios.

The consistent improvement across these metrics, particularly the convergence of precision and recall alongside the sustained rise in mAP scores, highlights the YOLO-CCA model’s ability to effectively detect objects with high reliability and accuracy. These results validate the architectural choices and training strategies employed, showcasing the YOLO-CCA as a potent solution for object detection tasks within complex environments like poultry farms.

### 4.2. Results of the Threshold Processing Algorithm

**Figure 11:**
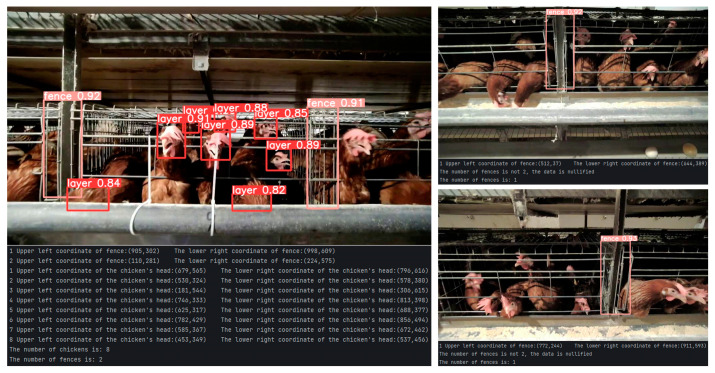
Visualization of threshold processing outcomes: selection of valid samples followed by chickens within the valid region.

Upon completing the object detection and acquiring relevant information about the chickens and the fence, we implemented a specific threshold-processing algorithm to more accurately determine the number of chickens inside the cage. The specific output results of this processing can be seen in Figure 11.

As observed on the left side of Figure 11, although the system identified 11 chickens overall, three of them were not located within the valid area defined by the fence. As a result, they were excluded from the final count, yielding an actual count of 8 chickens. The example on the right side of the figure demonstrates that since only one fence segment was recognized, it is not possible to ascertain the status of the entire cage. In such cases, the data were deemed invalid, and the step of counting chickens inside the cage was skipped.

From Table 5, the following observations can be made.

a. Sample selection rate: Both the YOLO-CCA and YOLOv8s algorithms achieved a selection rate of over 50%. The sample selection rate of YOLO-CCA rate is 1.2% higher than that of YOLOv8s. This suggests that when using YOLO-CCA, relatively more original data samples are considered valid and are selected for further analysis.

b. Chicken selection rate: The selection rates of both algorithms are close to 40% for chicken detection, with only a 0.9% difference between that of YOLO-CCA and that of YOLOv8. This indicates a comparable accuracy between the two algorithms in chicken selection.

Additionally, to assess performance under occlusion conditions, the experiments were conducted in an actual poultry farming environment across two layers of chicken cages, from bottom to top, with 40 cages per layer, totaling 80 cages. The experiments compared the actual counting numbers with the results from two algorithms: YOLOv8s single-frame detection at the center of the cages and our multi-frame continuous counting algorithm YOLO-CCA. The results are presented in Table 6.

The experiments showed that in the first layer, due to insufficient lighting, the recognition rates of both algorithms were lower than in the second layer. However, YOLO-CCA outperformed YOLOv8s in both layers. Particularly in the first layer with limited lighting, the chicken recognition rate of YOLO-CCA significantly increased by 13.9%, from 74.6% to 88.5%. Similarly, in the second layer, the recognition rate of YOLO-CCA improved by 12.5%, from 80.7% to 93.2%. These results highlight the effectiveness of the YOLO-CCA algorithm in addressing occlusion issues.

In summary, by using the threshold processing algorithm, we can more accurately filter and count the number of chickens inside the cage, ensuring the accuracy and validity of the data.

### 4.3. Performance Evaluation After Algorithm Deployment

While our current detection model meets the requirements of our single-camera inspection task, considering our future adoption of a multi-camera detection, we believe there is a need to further optimize the model to adapt to more complex scenarios. To ensure the efficiency and compatibility of the model in practical applications, we first converted it into the universal ONNX format. Subsequently, we transformed it from the ONNX format to the TensorRT model and successfully deployed it on the Jetson AGX Orin industrial computer onboard the robot. This industrial control machine, based on the arrch64 architecture, is specifically configured for deep learning computational power, boasting 275 TOPS of performance, making it well-suited for algorithm deployment. The entire deployment process performed well on the industrial control machine. The data performance after model deployment is presented in Table 7:

The data comparison before and after deployment reveals that although the model size increased post-deployment (attributed to TensorRT’s unique encapsulation format) and the recognition rate slightly declined, there was a significant enhancement in its operational speed to 90.9 FPS. This result attests to the efficacy of our deployment strategy and highlights the model’s immense potential in real-world applications.

It is worth noting that deploying the TensorRT model does not currently incorporate a function to measure overall accuracy; we primarily use the chicken recognition rate as our core evaluation metric. The exact overall target accuracy (AP_50:95_) will be a direction for our future optimization experiments.

In summary, the improvements achieved by the YOLO-CCA model have clear practical implications for poultry farming. The enhanced detection accuracy, higher *F1* score, and faster processing speed directly translate to more reliable and real-time chicken counting in caged environments. These improvements help reduce labor costs and counting errors while enabling timely management decisions on the farm. The reduction in model size and efficient performance also support deployment on low-power industrial devices, making the system well-suited for integration into robotic inspection platforms used in poultry farms.

## 5. Discussion

This research presents the YOLO-CCA model for detecting and counting live chickens in caged environments. Unlike previous approaches that primarily target free-range conditions or rely on static inspection methods [25,26,27], YOLO-CCA integrates the RevCol backbone and CoordATT attention mechanism into the YOLOv8s framework. This integration leads to improvements in accuracy, recall, and *F1* scores, particularly under challenging conditions with occlusion and environmental noise that are common in poultry farms. The experimental results demonstrate that YOLO-CCA outperforms both the original YOLOv8s and alternative variants employing different attention modules and backbone networks.

A central innovation of this work is the multi-frame threshold processing algorithm, which leverages video streams from robotic inspections to segment individual cages and update chicken counts in real time. This approach addresses limitations inherent in single-frame detection methods by reducing counting errors and facilitating prompt intervention when chicken numbers deviate from expected levels. In comparison to traditional manual counting methods, YOLO-CCA offers an automated solution that directly supports efficient farm management and proactive animal welfare monitoring.

Despite these advancements, the experiments have delineated certain limitations that the YOLO-CCA algorithm needs to address using the following criteria.

a. Lighting conditions: In practical poultry farm settings, lighting is primarily positioned above the cages. The experiments conducted have shown that the recognition rate for chickens in the bottom layer is generally lower due to these lighting challenges. To enhance the model’s robustness under varied lighting conditions, future iterations of this project may consider integrating camera hardware with supplementary lighting effects or employing image brightness pre-processing algorithms.

b. Localization of chicken cages: The introduction of the threshold processing algorithm further refines the model’s precision by selecting chickens within effective areas, avoiding background interferences and miscounting. Yet, the current localization approach relies primarily on timestamps to ascertain cage positions, which may be limited by the precision of the robot’s positioning. Future work will explore the use of more advanced spatial positioning technologies, such as combining QR codes or robotic coordinate data, to improve accuracy and reliability of localization.

c. Model deployment: The study successfully converted the YOLO-CCA model into TensorRT format and completed deployment on the Jetson AGX Orin industrial computer, enhancing processing speed for potential real-time monitoring applications. However, an increase in model size and a slight decline in recognition rate were observed post TensorRT deployment, necessitating a balance between model size, speed, and accuracy in future optimization efforts.

d. Accuracy improvement: Occlusion experiments on 80 chicken cages in real farming environments indicated that, while the current model has made significant progress in chicken counting, it still falls short of achieving completely accurate counts, particularly when there are too many chickens in a cage, leading to the possibility of undercounting. Therefore, further optimization of the algorithm is necessary to improve counting accuracy under various conditions. Moreover, we will establish rules for robotic rechecking to improve the accuracy of counting.

e. Limited data collection Time: Due to potential interference with the enterprise’s normal operations, data collection was limited to the morning hours and no repeated experiments were conducted. However, chicken behavior may vary across different times of the day, which could influence detection performance. In future work, we will collect data at various times and conduct repeated experiments to ensure the robustness and generalization of the algorithm.

**Figure 12:**
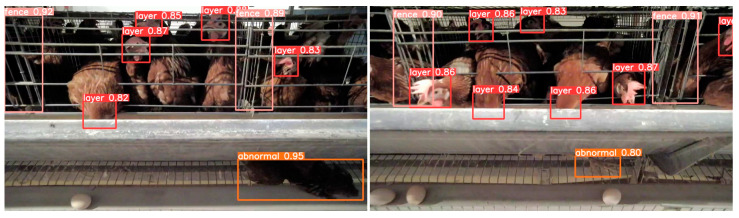
Detection Examples of Abnormal Conditions in Caged Chickens Using the YOLO-CCA Model.

Additionally, the ultimate goal for live chicken monitoring is to provide an accurate, real-time dynamic surveillance mechanism to optimize farm management. This mechanism, through periodic uploading of cage counting data to the cloud database, enables managers to instantly view data and monitor fluctuations in chicken numbers, especially detecting potential decreases due to deceased chickens. Building on this, our model also experimented with detecting anomalous chickens, showing promise in preliminary tests, as illustrated by the case example in Figure 12. However, the current model’s recognition of anomalies is limited to a small sample size. Thus, expanding the repository of such samples will be essential to develop a more robust anomaly detection system.

The scientific impact of this work lies in its integration of advanced deep learning techniques. By directly comparing YOLO-CCA with previous methods and demonstrating its performance in real-world agricultural environments, this study provides insights into the optimization of computer vision systems for precision livestock farming.

## 6. Conclusions

This study proposes the YOLO-CCA method, which integrates the CoordATT mechanism and the RevCol backbone to improve the accuracy and efficiency of live chicken counting in caged environments. Utilizing a multi-frame threshold processing algorithm, it accurately counts chickens per cage during robotic inspections and transmits this information to a cloud database. The model optimized through TensorRT has been deployed on the Jetson AGX Orin industrial computer, achieving real-time monitoring capabilities.

Future work should further improve the algorithm’s accuracy and enhance its robustness against varying lighting conditions and other environmental factors. Optimizing model parameters could lead to even better performance and efficiency. Advancements in the automation and intelligence levels of the live chicken counting system are also planned, aiming to create a more autonomous and adaptive solution. The system is capable of obtaining the daily count of chickens per cage through multiple robotic inspections. If there is a discrepancy between the counted number and the initial manual count, the system can pinpoint which area of the coop is experiencing issues for prompt resolution, thus ensuring breeding efficiency and poultry welfare. Additionally, exploring advanced data analysis techniques, such as integrating predictive analytics or machine learning models for anomaly detection, is necessary to enhance the system’s proactive management capabilities.

In summary, this study not only provides an effective poultry management tool for caged environments but also demonstrates the potential and application prospects of deep learning technology in agricultural automation. This research is of significant importance for optimizing modern livestock management, improving production efficiency, and ensuring animal welfare.

## Figures and Tables

**Table 1:** Hyperparameter definitions for the object detection model.

Hyperparameters	Object Detection Model
Batch size	32
Epochs	300
Iteration	60,938
Learning rate	1 × 10^−4^
Num of iteration	203
Num of workers	4
Optimizer	SGD

**Table 2:** Comparative performance analysis of YOLOv8s with different attention mechanisms.

Algorithms	Experimental Results						
	*P* ^1^	*R* ^2^	*F1* score	AP_50:95_^3^	Speed (FPS ^4^)	Parameters	Model Size (MB ^5^)
**YOLOv8s**	92.5%	94.9% ↑	93.7%	77.8%	66.6 ↑	11,126,745 ↓	21.97 ↓
**YOLOv8s+SA**	96.5% ↑	92.8%	94.6%	78.9%	50.0	11,127,273	21.98
**YOLOv8s+EMA**	96.4%	93.3%	94.8%	79.1% ↑	45.5	11,222,169	22.17
**YOLOv8s+SGE**	95.4%	94.2%	94.8%	79.0%	50.0	11,126,809	21.98
**YOLOv8s+SimAM**	96.1%	93.3%	94.7%	78.2%	52.6	11,126,745 ↓	21.97 ↓
**YOLOv8s+CoordATT**	96.0%	94.0%	95.0 %↑	79.1% ↑	52.6	11,188,073	22.11

^1^ *P* = Precision; ^2^ *R* = Recall; ^3^ AP_50:95_ = Average Precision for IoU values ranging from 0.5 to 0.95; ^4^ FPS *=* Frame Per Second; ^5^ MB = MByte. The best values within the detection model are underlined with arrows.

**Table 3:** Comparative performance analysis of YOLOv8s with the CoordATT attention mechanism across different backbone substitutions.

Algorithms	Experimental Results						
	*P*	*R*	*F1* score	AP_50:95_	Speed (FPS)	Parameters	Model Size (MB)
**YOLOv8s**	92.5%	94.9%	93.7%	77.8%	66.6 ↑	11,126,745	21.97
**YOLOv8s+CoordATT+LSKNet**	96.2%	96.4%	96.3%	79.4%	20.0	10,366,359	20.63
**YOLOv8s+CoordATT+RevCol**	97.4% ↑	96.0%	96.7 % ↑	80.6% ↑	33.3	8,268,009 ↓	16.50 ↓
**YOLOv8s+CoordATT+Resnet18**	95.9%	96.5%	96.2%	80.3%	43.5	17,946,025	35.30
**YOLOv8s+CoordATT+Fasternet**	96.9%	96.5%	96.7 % ↑	80.1%	31.3	8,678,317	17.22
**YOLOv8s+CoordATT+** **ConvNeXtV2**	96.2%	96.9% ↑	96.5%	80.0%	29.4	10,170,617	20.10
**YOLOv8s+CoordATT+** **EfficientFormerV2**	95.3%	96.7%	96.0%	79.9%	23.8	9,527,569	49.11

The best values within the detection model are underlined with arrows.

**Table 4:** Comparative evaluation of YOLOv8 variants in ablation experiments and benchmarking against other algorithms.

Algorithms	Experimental Results						
	*P*	*R*	*F1* score	AP_50:95_	Speed (FPS)	Parameters	Model Size (MB)
**YOLOv8s**	92.5%	94.9%	93.7%	77.8%	66.6 ↑	11,126,745	21.97
**YOLOv7-tiny**	96.9%	93.4%	95.1%	74.7%	62.5	6,013,008 ↓	11.99 ↓
**YOLOX-s**	94.6%	87.1%	90.7%	76.0%	33.3	8,940,000	70.16
**YOLOv8s+CoordATT**	96.0%	94.0%	95.0%	79.1%	52.6	11,188,073	22.11
**YOLOv8s+RevCol**	96.0%	97.1% ↑	96.5%	80.0%	37.0	8,206,681	16.36
**YOLOv8s+CoordATT+RevCol**	97.4% ↑	96.0%	96.7% ↑	80.6% ↑	33.3	8,268,009	16.50

The best values within the detection model are underlined with arrows.

**Table 5:** Comprehensive comparative analysis of chicken detection outcomes before and after implementing the threshold processing algorithm for the YOLOv8s and YOLO-CCA models.

Experimental Results	Algorithms	
	YOLOv8s	YOLO-CCA
**Initial sample size**	650	650
**Actual chicken count**	4017	4017
**Detected chicken count**	3733	3838 ↑
**Sample count after threshold processing**	336	344 ↑
**Chicken count after threshold processing**	1480	1554 ↑
**Sample selection rate**	51.7%	52.9% ↑
**Chicken selection rate**	39.6%	40.5% ↑

The best values within the detection model are underlined with arrows.

**Table 6:** Comparison of the performance of multi-frame and single-frame detection algorithms under actual chicken breeding conditions.

Experimental Results (Both Apply the Thresholding Algorithm)	Location		
	The First Layer	The Second Layer	Total
**Actual chicken count**	244	249	493
**Chicken count—YOLOv8s**	182	201	383
**Chicken count—YOLO-CCA**	216 ↑	232 ↑	448 ↑
**Chicken recognition rate (** **YOLOv8s** **)**	74.6%	80.7%	77.7%
**Chicken recognition rate (** **YOLO-CCA** **)**	88.5% ↑	93.2% ↑	90.9% ↑

The best values within the detection model are underlined with arrows.

**Table 7:** Comparative analysis of the algorithm’s performance before and after deployment.

Algorithms	Experimental Results		
	Chicken Recognition Rate	Speed (FPS)	Model Size (MB)
**YOLOv8s**	92.9%	66.6	21.97
**YOLOv8s (TensorRT)**	92.7%	100.0 ↑	60.0
**YOLO-CCA**	95.5% ↑	33.3	16.50 ↓
**YOLO-CCA (TensorRT)**	93.2%	90.9	58.2

The best values within the detection model are underlined with arrows.

## Data Availability

The data presented in this study are available on request from the corresponding author.
